# Clinical analysis of 96 patients with intraorbital foreign bodies: A 10-year retrospective study

**DOI:** 10.3389/fmed.2022.1018905

**Published:** 2022-11-16

**Authors:** Yayan You, Xinghua Wang, Shengnan Cheng, Ru Zhu, Bowen Wang, Shuang Li, Fagang Jiang

**Affiliations:** ^1^Department of Ophthalmology, Union Hospital, Tongji Medical College, Huazhong University of Science and Technology, Wuhan, China; ^2^Department of Ophthalmology, Wuhan Hospital of Traditional Chinese and Western Medicine, Wuhan, China; ^3^Department of Ophthalmology, The Central Hospital of Wuhan, Tongji Medical College, Huazhong University of Science and Technology, Wuhan, China

**Keywords:** orbit, foreign body, trauma, CT, MRI

## Abstract

**Introduction:**

To investigate the clinical manifestations, diagnosis, and surgical treatment of intraorbital foreign bodies (IOFBs).

**Methods:**

Patients with IOFBs were enrolled from Wuhan Union Hospital between January 2011 and January 2021. Demographic and clinical information was extracted, including gender, age, cause and entrance of the trauma, material, size and quantity of foreign body, visual function, ocular complications, imaging findings, and surgical intervention. The patients were divided into two groups according to the timeline, group A (from January 2011 to December 2015, *n* = 39) and group B (from January 2016 to January 2021, *n* = 57).

**Results:**

The 96 patients (81 men and 15 women) were enrolled in this series, with a median age of 39.5 (1.6–76.0) years. Work-related injuries were the cause of IOFBs in 45 individuals (46.9%). Three patients (3.3%) presented severe visual impairment, and 39 patients (42.4%) presented blindness. The majority of foreign bodies were metal (44.8%), followed by wood (26.0%). Computed tomography (CT) and magnetic resonance imaging (MRI) were performed, respectively, on 89 (92.7%) and 21 (21.9%) patients with IOFBs, in which the detection rate was 80.9% for CT and 81.0% for MRI. Among the 25 patients with intraorbital wooden foreign bodies (IOWFBs), the utilization and detection rates of MRI were 50.0% and 40.0% in group A, and 93.3% and 92.9% in group B, with significant differences in both rates between the two groups (both *P* < 0.05). The IOWFBs detection rate in MRI was significantly higher than that in CT (78.9% vs. 45.8% overall and 92.9% vs. 53.5% in group B). The detection rates of IOFBs and IOWFBs in initial surgery were statistically different between the two groups, of which the rates were 84.6% and 40.0% in group A and 98.2% and 93.3% in group B. The reoperation rate of IOWFBs in group B (20.0%) was significantly lower than that in group A (70.0%).

**Conclusion:**

IOFBs were mainly caused by work-related injuries and might lead to serious visual impairment. The application and detectability of MRI in IOWFBs improved in recent years, and MRI presented better detectability than CT in diagnosing IOWFBs. Thus, MRI should be recommended despite negative CT findings.

## Introduction

Intraorbital foreign bodies (IOFBs) are severe and complicated ocular trauma, constituting 16.7% of orbital injuries (1, 2). It is defined as the foreign bodies located in the orbital cavity, behind the orbital septum and the eyeball (3), which can cause damage to vision, eyeball, optic nerve, vasculature, and extraocular muscles (4, 5). Besides, IOFBs can cause chemical toxicity and microbial infection (6) and invade extra orbital tissue (7). IOFBs are more common in young men and are typically caused by a high-velocity or a relatively trivial orbital injury (8).

Most of IOFBs are metal, and others include wood, glass, stone, plastic, etc. (9). Orbital imaging is indispensable in diagnosing IOFBs, in which computed tomography (CT) is the first choice to find and locate IOFBs, especially for high-density foreign bodies. On the other hand, magnetic resonance imaging (MRI) cannot be performed until metal foreign bodies are excluded. Compared with other foreign bodies, the diagnosis of intraorbital wooden foreign bodies (IOWFBs) can be particularly difficult due to the limitations of medical history and external signs, as well as the initial negative performance of CT (9). As a result, IOWFBs may be overlooked in the initial stage of injury, resulting in severe orbital tissue necrosis and intraorbital infection, such as orbital cellulitis, abscess formation, and orbital fistula (4, 10).

According to the treatment principle, it has been recommended that IOFBs should be surgically removed, except for blunt inorganic, asymptomatic, or deep-seated IOFBs (3, 11, 12). As the size, material, chemical activity, and location of IOFBs differ dramatically, the manifestation and surgical approaches may also vary. In practice, IOFBs and surgical approaches may affect visual function, eye movement, and appearances, such as proptosis, enophthalmos, and eyelid malformations. Therefore, it has been recommended to pay particular attention to the protection of the optic nerve, extraocular muscles, and other vascular and soft tissues, which could be beneficial for retaining vision, eye movement, and other ocular functions (4, 10). Nonetheless, due to the negative CT performance and fragility of some special IOFBs, especially IOWFBs, missed diagnosis and residual often happen during the initial surgery. While ophthalmologists have been interested in diagnosing IOFBs (13), there are insufficient studies focused on the detection rate and the surgery guidance of IOFBs. In the present study, we analyzed the clinical manifestations, applications of CT/MRI, and surgical interventions of IOFBs over 10 years to help manage IOFBs.

## Methods

### Study design and participants

This retrospective study was approved by the Ethics Committee of Wuhan Union Hospital (UHCT22320) and complied with the principles of the Declaration of Helsinki. The electronic medical records of inpatients with IOFBs confirmed by surgery from January 2011 to January 2021 were reviewed. Patients meeting any of the following criteria were excluded from the study: (1) patients with incomplete medical records and non-IOFB cases such as conjunctival, corneal, or intraocular foreign bodies; (2) patients suffering from corneal diseases, lens diseases, glaucoma, and other eye diseases that may affect the results of pre-traumatic vision tests; (3) patients with a history of other ocular surgery or trauma; and (4) patients with systemic diseases such as severe diabetes, mental disorders, and neurological diseases.

### Data collection

We extracted the following demographic and clinical information from each patient: gender, age, ocular laterality, cause and entrance of the trauma, material, size and quantity of foreign body, visual function, ocular complications, imaging findings, and surgical intervention. Most patients underwent imaging examinations, including orbital CT, MRI, X-ray, or B-ultrasound. MRI scans were used for patients suspected of having IOWFBs, while CT excluded metal foreign bodies. According to the size and location of IOFBs, different surgical approaches were applied to patients, such as lateral or anterior orbitotomy through a cutaneous or conjunctival approach. All patients were treated with systemic antibiotics after surgery.

### Grouping and grading

We divided the patients into two groups based on the timeline to determine if the detection rate of IOFBs altered as our understanding of IOFBs, imaging technology, and clinical experiences evolved. The patients with IOFBs admitted to this hospital from January 2011 to December 2015 were defined as group A (*n* = 39) and from January 2016 to January 2021 were group B (*n* = 57).

We graded the visual acuity (VA) concretely, where grade 1 indicated vision ≧ 20/40; grade 2 indicated vision <20/40 to ≧ 20/200; grade 3 indicated vision <20/200 to ≧ 20/400; grade 4 indicated vision count finger (CF) to <20/400, and grade 5 indicated hand motion (HM), light perception (LP), and no light perception (NLP). Based on the criteria of the World Health Organization (14–17), we defined grade 3 as severe visual impairment, while grades 4 and 5 as blindness.

### Statistical analysis

Statistical analysis was performed using SPSS software (version 26.0) (IBM Corp., Armonk, NY, USA). Continuous data were represented by median (interquartile), and categorical data were represented by n (%). For continuous variables, the Shapiro–Wilk test and Levene's test were used to test the normal distribution and homogeneity of variance. Then, a Mann-Whitney *U* test was used to compare the data between groups. Associations between the other variables were analyzed using the Continuity correction, Chi-squared, or Fisher's exact test. *P* < 0.05 was considered statistically significant.

## Results

### Characteristics of the participants

The 96 patients with IOFBs were predominantly young, with a median age of 39.5 (25% IQR: 27.5, range 1.6–76) years. Males were more significantly affected than females, with a ratio of 5.4:1 (81 men, 15 women). The comparison of different variables between the two groups is shown in Table 1, and there was no significant difference in age, gender, and ocular laterality between the two groups.

**Table 1:** Clinical characteristics of the 96 patients with intraorbital foreign bodies.

	**All (*N* = 96)**	**Group A (*N* = 39)**	**Group B (*N* = 57)**	***P***
**Age (years) (median)**	39.5 (27.5–50.0)	43.0 (16.0–49.0)	38.0 (28.0–51.0)	0.958^a^
**Sex (males, %)**	81 (84.4%)	33 (84.6%)	48 (84.2%)	0.957^b^
**Ocular laterality (right, %)**	50 (52.1%)	21 (53.8%)	29 (50.9%)	0.775^b^
**Cause of Injuries**				0.295^c^
Work Injuries	45 (46.9%)	19 (48.7%)	26 (45.6%)	
Assaults	13 (13.5%)	3 (7.7%)	10 (17.5%)	
Falls	10 (10.4%)	5 (12.8%)	5 (8.8%)	
Fireworks	12 (12.5%)	7 (17.9%)	5 (8.8%)	
Traffic accidents	12 (12.5%)	5 (12.8%)	7 (12.3%)	
Others	4 (4.2%)	0 (0.0%)	4 (7.0%)	
**Traumatic entrance**				0.920^c^
Eyelid	66 (68.8%)	28 (71.8%)	38 (66.7%)	
Conjunctiva	27 (28.1%)	10 (25.6%)	17 (29.8%)	
Others	3 (3.1%)	1 (2.6%)	2 (3.5%)	
**Presenting symptoms**				0.295^c^
Eyelid injuries	66 (68.8%)	25 (64.1%)	41 (71.9%)	
Orbital fracture	34 (35.4%)	13 (33.3%)	21 (36.8%)	
EOM restriction/strabismus	28 (29.2%)	11 (28.2%)	17 (29.8%)	
Conjunctival injury	27 (28.1%)	12 (30.8%)	15 (26.3%)	
Penetrating injury of the eyeball	26 (27.1%)	10 (25.6%)	16 (28.1%)	
Exophthalmos	9 (9.4%)	4 (10.3%)	5 (8.8%)	
Orbital infection	22 (22.9%)	9 (23.1%)	13 (22.8%)	

EOM, Extraocular movement.

a Mann–Whitney U-test;

b Chi-squared test;

c Fisher's Exact Test.

Injuries sustained by patients with IOFBs were caused by work-related incidents (45/96, 46.9%), assaults (13/96, 13.5%), falls (10/96, 10.4%), fireworks (12/96, 12.5%), traffic accidents (12/96, 12.5%), and others (4/96, 4.2%). The most common original traumatic entrance was the eyelid (66/96, 68.8%), followed by the conjunctiva (27/96, 28.1%). Sixty-six patients (68.8%) presented eyelid injuries (including eyelid laceration, ptosis, and hypophysis), 34 patients (35.4%) presented orbital fracture, 28 patients (29.2%) presented extraocular movement restriction and strabismus, 27 patients (28.1%) presented conjunctival injury, 26 patients (27.1%) presented combined penetrating injury of the eyeball, nine patients (9.4%) presented proptosis, 22 patients (22.9%) presented orbital infection (including orbital fistula, cellulitis, mass, and abscess) (Table 1).

Except for four children who could not cooperate with the examination, the preoperative visual acuity of 92 patients (92/96, 95.8%) was recorded. Among these patients, 34 patients (34/92, 37.0%) presented grade 1 visual acuity, and 16 (16/92, 17.4%) presented grade 2. Meanwhile, severe visual impairment (grade 3) and blindness (grades 4 and 5) were presented in 3 (3/92, 3.3%) and 39 (39/92, 42.4%) of the patients, respectively.

### Materials and quantities of foreign bodies

**Figure 1:**
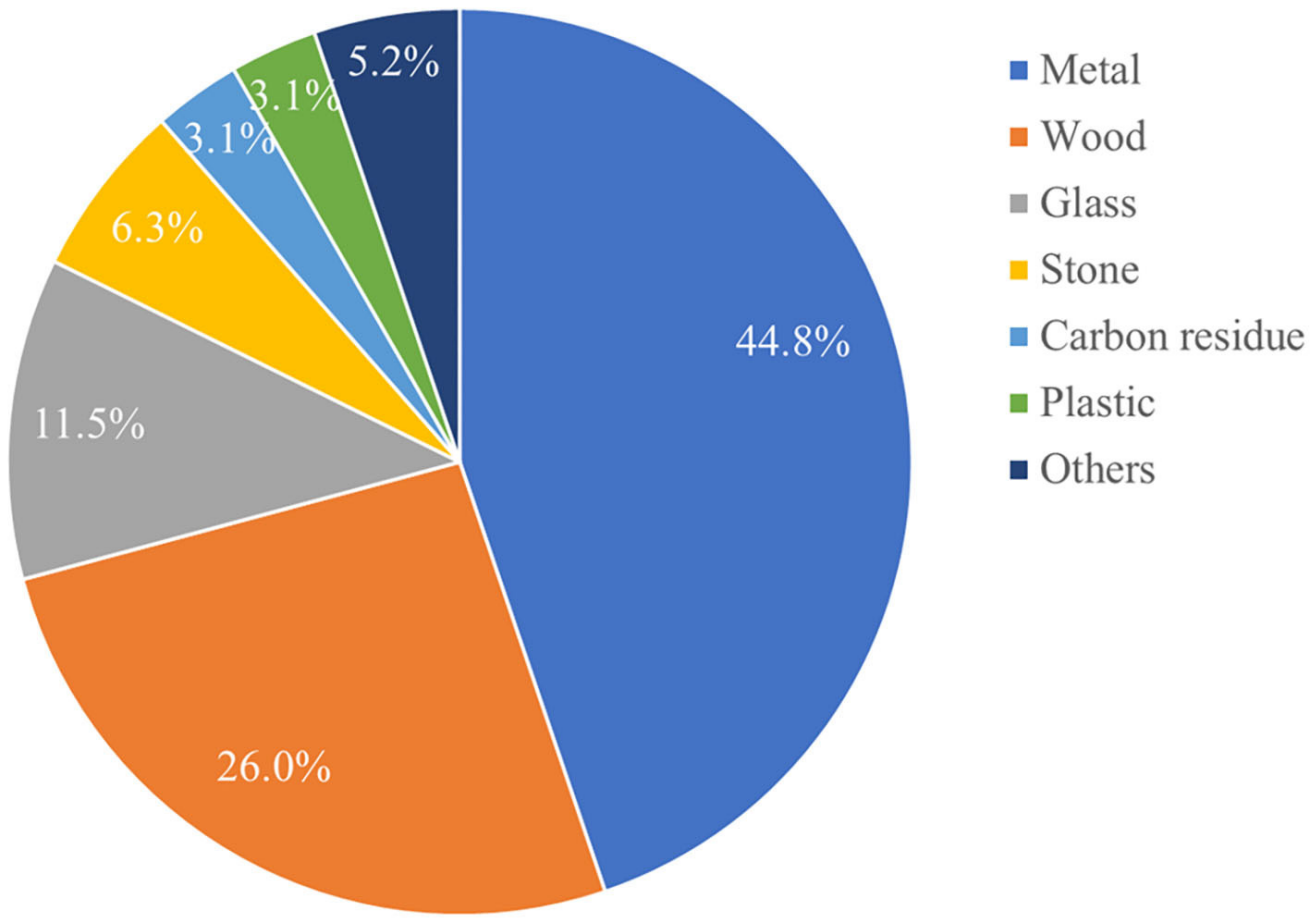
The materials of foreign bodies (in %).

The majority of foreign bodies were metal 44.8% (43/96), followed by wood 26.0% (25/96), glass 11.5% (11/96), stone 6.3% (6/96), carbon residue 3.1% (3/96), and plastic 3.1% (3/96; Figure 1). In addition, the maximum length of foreign bodies was 9.2 cm, and the maximum number of foreign bodies removed in one operation was 27.

### Computerized tomography

Except for seven patients, 92.7% (89/96) patients underwent orbital CT, of which 80.9% (72/89) were found foreign bodies. The utilization and detection rates in group A were 92.3% (36/39) and 77.8% (28/36), while those in group B were 93.0% (53/57) and 83.0% (44/53), respectively. Among the foreign bodies indicated by CT, high-density foreign bodies accounted for 75.0% (54/72), mostly metal foreign bodies (75.9%, 41/54); the remaining 25.0% (18/72) were medium- and low-density foreign bodies, mostly wooden foreign bodies (61.1%, 11/18). Therefore, we analyzed these two representative foreign bodies (metal and wood). Of the 43 patients with metal IOFBs, the utilization and detection rates of CT were 95.3% (41/43) and 100.0% (41/41), both 100.0% (17 /17) in group A, and 92.3% (24/26) and 100.0% (24/24) in group B. Of the 25 patients with IOWFBs, the utilization and detection rates of CT were 96.0% (24/25) and 45.8% (11/24), 90.0% (9/10) and 33.3% (3/9) in group A, and 100.0% (15/15) and 53.5% (8/15) in group B. There was no statistical difference in the utilization rate and detection rate of CT in IOFBs between the two groups (Table 2).

**Table 2:** The findings of imaging examination and surgery in patients with intraorbital foreign bodies.

	**ALL**	**Group A**	**Group B**	***P***
**Received computerized tomography**			
**Overall IOFBs**	*n* = 96	*n* = 39	*n* = 57	
Utilization rate	92.7% (89/96)	92.3% (36/39)	93.0% (53/57)	1.0^a^
Detection rate	80.9% (72/89)	77.8% (28/36)	83.0% (44/53)	0.537^b^
**Metal IOFBs**	*n* = 43	*n* = 17	*n* = 26	
Utilization rate	95.3% (41/43)	100.0% (17 /17)	92.3% (24/26)	0.511^c^
Detection rate	100.0% (41/41)	100.0% (17 /17)	100.0% (24/24)	
**IOWFBs**	*n =* 25	*n =* 10	*n =* 15	
Utilization rate	96.0% (24/25)	90.0% (9/10)	100.0% (15/15)	0.400^c^
Detection rate	45.8% (11/24)	33.3% (3/9)	53.5% (8/15)	0.423^c^
**Received magnetic resonance imaging**				
**Overall IOFBs**	*n =* 96	*n =* 39	*n =* 57	
Utilization rate	21.9% (21/96)	15.4% (6/39)	26.3% (15/57)	0.203^b^
Detection rate	81.0% (17/21)	50.0% (3/6)	93.3% (14/15)	0.053^c^
**IOWFBs**	*n =* 25	*n =* 10	*n =* 15	
Utilization rate	76.0% (19/25)	50.0% (5/10)	93.3% (14/15)	0.023^c^
Detection rate	78.9% (15/19)	40.0% (2/5)	92.9% (13/14)	0.037^c^
**Received surgical treatment**			
**Overall IOFBs**	*n =* 96	*n =* 39	*n =* 57	
Detection rate	92.7% (89/96)	84.6% (33/39)	98.2% (56/57)	0.034^a^
Missed diagnosis rate	7.3% (7/96)	15.4% (6/39)	1.8% (1/57)	
Residual rate	5.6% (5/89)	6.1% (2/33)	5.4% (3/56)	1.0^a^
Reoperation rate	12.5% (12/96)	20.5% (8/39)	7.0% (4/57)	0.099^a^
**IOWFBs**	*n =* 25	*n =* 10	*n =* 15	
Detection rate	72.0% (18/25)	40.0% (4/10)	93.3% (14/15)	0.007^c^
Missed diagnosis rate	28.0% (7/25)	60.0% (6/10)	6.7% (1/15)	
Residual rate	16.7% (3/18)	25.0% (1/4)	14.3% (2/14)	1.0^c^
Reoperation rate	40.0% (10/25)	70.0% (7/10)	20.0% (3/15)	0.034^c^

a Continuity correction;

b Chi-squared test;

c Fisher's exact test.

### Magnetic resonance imaging

There were 21.9% (21/96) IOFB patients who underwent orbital MRI, of which 81.0% (17/21) were found to have foreign bodies. The utilization and detection rates of MRI in group A were 15.4% (6/39) and 50.0% (3/6), while they were 26.3% (15/57) and 93.3% (14/15) in group B, respectively. IOWFBs accounted for a large proportion of 90.5% (19/21) among the 21 IOFBs patients who underwent MRI. Of the 25 patients with IOWFBs, MRI utilization and detection rates were 76.0% (19/25) and 78.9% (15/19). Separately, there were 10 patients with IOWFBs in group A and 15 patients with IOWFBs in group B. However, only five patients (50.0%, 5/10) underwent an MRI examination, and two (40.0%, 2/5) were positive for this condition in group A. Among the 15 patients with IOWFBs in group B, 14 patients (93.3%, 14/15) received an MRI examination, and 13 patients (92.9%, 13/14) presented positive images, which indicated a significantly higher utilization rate and detection rate than group A (both *P* < 0.05; Table 2).

### The comparison of CT and MRI in the diagnosis of IOWFBs

Our research showed no statistical difference in CT and MRI utilization of IOWFBs in the overall population or group A or B (all *P* > 0.05). The detection rates of CT and MRI were 45.8% and 78.9% in the overall population, 33.3% and 40.0% in group A, and 53.5% and 92.9% in group B. There were significant differences in the detection rates of overall and group B between the two techniques (for CT vs. MRI in overall, *P* = 0.027; for CT vs. MRI in group A, *P* = 1.0; and for CT vs. MRI in group B, P = 0.035; Table 3).

**Table 3:** The comparison of CT and MRI in the diagnosis of intraorbital wooden foreign bodies.

	**Utilization rate**			**Detection rate**		
	**CT**	**MRI**	***P***	**CT**	**MRI**	***P***
**ALL**	96.0%	76.0%	0.103^a^	45.8%	78.9%	0.027^b^
**Group A**	90.0%	50.0%	0.141^c^	33.3%	40.0%	1.0^c^
**Group B**	100.0%	93.3%	1.0^c^	53.5%	92.9%	0.035^c^

a Continuity correction;

b Chi-squared test;

c Fisher's exact test.

### The absence of imaging

In this study, six patients (6.3%) were diagnosed directly by surgical exploration without CT or MRI. Interestingly, four patients (4.2%) were found negative in both CT and MRI images, but wooden foreign bodies were confirmed for all of them during the surgeries.

### Treatment strategy

All 96 patients were confirmed to have IOFBs by surgery, although some received multiple surgeries. The total rates of detection, missed diagnosis, and residue in the initial surgery and reoperation were 92.7% (89/96), 7.3% (7/96), 5.6% (5/89), and 12.5% (12/96), respectively. Separately, the corresponding rates were 84.6% (33/39), 15.4% (6/39), 6.1% (2/33), 20.5% (8/39) in group A, and 98.2% (56/57), 1.8% (1/57), 5.4% (3/56), 7.0% (4/57) in group B. There was a statistically significant difference in the detection rate of the initial surgeries between group A and group B (*P* = 0.034; Table 2).

Unlike metal foreign bodies, wooden foreign bodies are the main reason for missed diagnosis and residue of IOFBs. In the initial surgeries of 25 patients with IOWFBs, the detection rates of detection, missed diagnosis, residue, and reoperation were 72.0% (18/25), 28.0% (7/25), 16.7% (3/18) and 40.0% (10/25), respectively. Separately, the corresponding rates were 40.0% (4/10), 60.0% (6/10), 25.0% (1/4), 70.0% (7/10) in group A, and 93.3% (14/15), 6.7% (1/15), 14.3% (2/14), 20.0% (3/15) in group B. The detection rate of the initial surgery in group B was significantly higher than in group A (*P* = 0.007), while the rate of reoperation in group B was significantly lower than in group A (*P* = 0.034; Table 2).

## Discussion

Periorbital penetrating injury by foreign bodies can cause intraorbital foreign bodies (IOFBs) and seriously impair visual function, eye movement function, and ocular appearance. Complicated IOFBs, especially combined with orbital-cranial orbital-nasal penetrating injuries, could induce severe trans-disciplinary infection, hemorrhage, and even death. Nearly half (46.9%) of IOFBs were caused by work-related injuries in our study, which was consistent with the finding by Szabo B et al. (3). In our study, the most common IOFBs were metal (44.8%), the same as previous references (3, 4). Wooden foreign bodies (26.0%) also accounted for a large proportion, which might increase the difficulty of diagnosis and treatment. Additionally, atypical early clinical symptoms in some patients with trivial trauma or vague medical history often resulted in a misdiagnosis, missed diagnosis, and delayed or even improper treatment (8). Therefore, the diagnosis and management of IOFBs remain a challenge to ophthalmologists.

The clinical manifestations of IOFBs are complicated and varied, including periorbital redness and swelling, ptosis, proptosis, extraocular movement restriction, and strabismus, and may be accompanied by ocular penetrating injury or orbital fracture. Previous studies showed that the IOFBs surgery does not aggravate visual impairment (4, 11, 18). However, penetrating injury of the eyeballs caused by IOFBs could cause vision loss, which has been confirmed in this study. We found that 3.3% and 42.4% of the patients had severe visual impairment and blindness at the initial visit, respectively. Additionally, we have observed signs of orbital infection (orbital cellulitis, fistula, abscess, or mass) in some patients, which is a high alert of the possible presence or residue of intraorbital wooden foreign bodies (IOWFBs) (3, 12).

A detailed medical history and careful ophthalmic examination are necessary for patients with direct orbital stab wounds and suspected IOFBs (4, 8). Preoperative evaluation of the material, size, and location of IOFBs could improve the success of surgery (18–20). CT is the first choice for IOFBs (11, 21, 22), which can find concomitant orbital fracture, orbital hematoma, or abscess. In our study, we performed CT scans for 89 patients before surgical intervention, of which 72 (80.9%) were found with foreign bodies. Meanwhile, MRI scans were performed in 21 patients with suspected IOFBs to exclude metallic foreign bodies by CT, and foreign bodies were found in 17 patients (81.0%). Previous studies showed that CT scans allowed accurate detection and localization of high-density IOFBs like metal or glass (8, 23) and demonstrated that the metal artifacts help assess IOFB properties. We observed that the CT images of wooden foreign bodies varied after trauma: air bubbles in the acute stage, intraorbital fat in the subacute phase, and extraocular muscles in the chronic phase (3, 5, 24). Therefore, we believe that diagnosing low-density objects such as IOWFBs is challenging, and CT scans are not sufficient to detect small IOWFBs, especially in the initial stage.

Conversely, on both T1 and T2 weighted MRI sequences, wood characteristically demonstrates hypointense relative to intraorbital fat, regardless of water content (8, 25, 26). Additionally, the signal of the wooden foreign body in the T1-weighted image is more uniform, easier to distinguish (9, 26), and less likely to obtain motion artifacts during imaging than in the T2-weighted image (5, 27). Therefore, MRI, especially a T1-weighted image, is highly recommended when metal foreign bodies are excluded, or there is a clear history of wooden stab injuries.

Most previous studies have not described the detection and missed diagnosis rate of IOFBs. In the present study, we divided 10 years into two segments, January 2011–December 2015 (group A) and January 2016–January 2021 (group B). The results demonstrate that the utilization rate and detection rate of MRI in IOWFBs were significantly higher in the second 5-year period. Besides, we found that MRI had a considerably greater IOWFBs detection rate than CT. These results suggest that ophthalmologists should pay attention to the role of MRI in an IOWFB diagnosis, which can greatly reduce the possibility of a misdiagnosis or a missed diagnosis. Despite negative imaging results for both CT and MRI, IOWFBs were verified in four individuals during the surgical investigation, demonstrating that this diagnosis could not be ruled out.

However, while surgery is the standard method of treating IOFBs, there are circumstances in which surgery is not indicated. Wood's porous organic nature makes it an ideal breeding ground for bacteria and other germs that could cause infection if left untreated (5, 28, 29). It has been reported that more than half of patients with IOWFBs develop an orbital infection (9, 30, 31), necessitating the removal of all wooden foreign bodies. Large foreign bodies should also be removed surgically because they always cause eye movement disorders, oppressive symptoms, and visual dysfunction (3, 18). However, asymptomatic small foreign bodies like glass, stone, metal (except copper) or that located at the orbital apex can be retained without removal (8, 32), as the surgery itself may cause serious complications such as orbital bleeding or damage to important nerves and blood vessels (12, 18).

**Figure 2:**
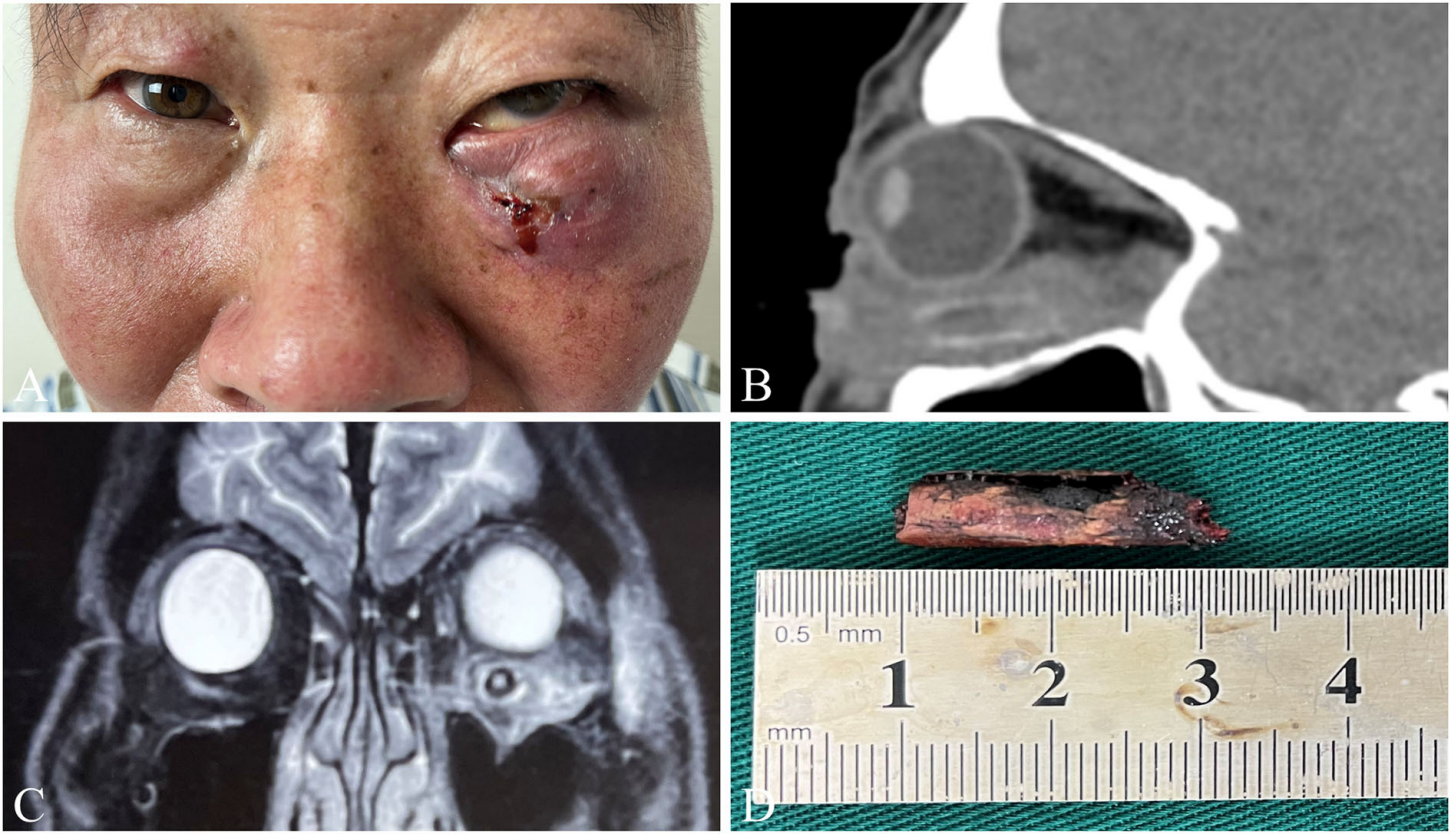
A representative case of IOWFBs. A 55-year-old man who suffered pain and swelling in his left eye after being injured by a branch and received two separate debridement operations to remove multiple branch fragments at a local hospital. A month after the second operation, he was transferred to our hospital for a thorough examination and treatment as his would worsened. **(A)** Since a fistula with mucinous secretions in the lower eyelid was exhibited, he was admitted to our hospital 1 month after the second operation. **(B)** The sagittal CT scan showed a high-density shadow under the left eyeball, with a size of about 20 × 6 mm. **(C)** MRI detected a hollow columnar shadow below the inferior rectus muscle of the left eye. **(D)** A branch ~20 mm long was taken out by exploratory surgery.

With an improved understanding of IOFBs, especially IOWFBs, and the development of imaging technology and clinical experiences, the detection rate of initial surgery has significantly improved in recent years. However, some patients with IOFBs received more than two surgeries due to the missed diagnosis or residual foreign bodies during the initial surgery (as shown in Figure 2). For all patients with IOWFBs, the reoperation rate was as high as 40.0%. Fortunately, the reoperation rate of IOWFBs significantly declined in the second 5-year period. Appropriate surgical strategies are essential for reducing the reoperation rate and improving treatment outcome. First, surgeries should be performed safely under general anesthesia, especially for patients with IOFBs in deep orbital positions, reoperation, and severe orbital injuries (18, 33). Secondly, an individual surgical incision should be designed according to the location of the IOFBs, including the common anterior and lateral approaches, and the relatively rare endoscopic transnasal and suprafrontal approaches (3). Consistent with the previous literature, our study found that the common original traumatic entrances in IOFBs patients were the eyelid (68.8%) and conjunctiva (28.1%). IOFBs located in the anterior orbit can be removed through the anterior approach of the original entrances, and the incision should be enlarged appropriately if necessary. Because IOWFBs may present specific infection manifestations, we can choose the top of the fistula, granuloma, or abscess for the initial surgical incision. The deep IOFBs should be removed through the lateral approach. In addition, when the IOFBs extend to extra orbital tissue, such as the paranasal sinus and cranial cavity, we should consult a neurologist and otolaryngologist. Finally, since some IOWFBs may break into several segments or debris, continued meticulous exploration is recommended even if an incomplete foreign body is found, which can help to avoid foreign body residue, and if there is a fistula or an abscess, complete removal of necrotic tissue, antibiotic saline irrigation of the wound, and placement of drainage strips are advised.

## Limitation

There were some limitations to our study. In this single-center retrospective study, a smaller sample size, selection bias, and variations among observers during the clinical analysis might exist. Another limitation of this study was that it covered a longer period of time, during which many patients lost contact and were unable to identify their final vision. Therefore, a multi-center study with larger populations and a prospective study (34) should be required to achieve a more comprehensive profile of IOFBs.

## Conclusion

IOFBs mainly occur in young and middle-aged men, mostly in work-related injuries, which could lead to serious visual impairment. Most of the foreign bodies were metal and wood. Although most foreign bodies could be detected by imaging and surgery, the missed diagnosis of IOWFBs could not be ignored. CT was considered the first choice for most IOFBs, but MRI had a significantly higher detection rate of IOWFBs. Thus, MRI should be recommended despite negative CT findings. The development of imaging technologies and clinical experiences help improve the detectability of IOWFBs and decrease the rate of missed diagnosis or reoperation.

## Data availability statement

The raw data supporting the conclusions of this article will be made available by the authors, without undue reservation.

## Ethics statement

Written informed consent was obtained from the individual(s) for the publication of any potentially identifiable images or data included in this article.

## Author contributions

FJ and SL designed the study. YY and XW collected data, performed the analyses, and drafted the manuscript. SC assisted in data statistics. FJ, SL, and YY contributed to the discussion and revised the manuscript. RZ and BW assisted in proofreading and revising the draft. All authors read and approved the final manuscript.

## Funding

This work was supported by the National Natural Science Foundation of China (No. 81900912) and the Natural Science Foundation of Hubei Province (No. 2021CFB524).

## Conflict of interest

The authors declare that the research was conducted in the absence of any commercial or financial relationships that could be construed as a potential conflict of interest.

## Publisher's note

All claims expressed in this article are solely those of the authors and do not necessarily represent those of their affiliated organizations, or those of the publisher, the editors and the reviewers. Any product that may be evaluated in this article, or claim that may be made by its manufacturer, is not guaranteed or endorsed by the publisher.
